# Proapoptotic Activity of Propolis and Their Components on Human Tongue Squamous Cell Carcinoma Cell Line (CAL-27)

**DOI:** 10.1371/journal.pone.0157091

**Published:** 2016-06-09

**Authors:** Urszula Czyżewska, Katarzyna Siemionow, Ilona Zaręba, Wojciech Miltyk

**Affiliations:** 1 Department of Pharmaceutical Analysis, Medical University of Bialystok, Bialystok, Poland; 2 Department of Medicinal Chemistry, Medical University of Bialystok, Bialystok, Poland; University of Sassari, ITALY

## Abstract

Propolis has been used since ancient times in folk medicine. It is a popular medicine possessing a broad spectrum of biological activities. This material is one of the richest sources of polyphenolic compounds such as flavonoids and phenolic acids. The ethanolic extract of propolis (EEP) evokes antibacterial, antiviral, antifungal and anticancer properties. Due to pharmacological properties it is used in the commercial production of nutritional supplements. In this study, gas chromatography coupled with mass spectrometry (GC-MS) was used to quantify main polyphenols in EEPs. The effect of EEPs, individual EEPs components (chrysin, galangin, pinocembrin, caffeic acid, *p*-coumaric acid, ferulic acid) and their mixture on viability of human tongue squamous cell carcinoma cell line (CAL-27) as well as the molecular mechanisms of the process were examined. The results of MTTs assay demonstrated that EEP, polyphenols and mixture of polyphenolic compounds were cytotoxic for CAL-27 cells in a dose dependent manner. The mechanism of cytotoxicity induced by these components undergoes through apoptosis as detected by flow cytometry. The ethanolic extracts of propolis activated caspases -3, -8, -9. Mixture of polyphenols was found as the most potent inducer of apoptosis thorough both intrinsic and extrinsic pathway. Therefore, we suggest that anticancer properties of propolis is related to synergistic activity of its main components.

## Introduction

Surgery and radiotherapy are the two principal methods used to treat tongue cancer. Both of therapies cause side effects, lower the quality of life and ultimately contributes to death [1, 2]. The death rate for these cancers has been increasing over the last 30 years [3]. Therefore, new chemopreventive and chemotherapeutic approaches for treatment of oral cancers are required.

Propolis is a resinous substance collected by honeybees from buds and exudates of various plants [4]. This natural product has been used in folk medicine since ancient times as an antibacterial and anti-inflammatory agent [5]. It has been demonstrated a broad spectrum of biological activities, such as antifungal, antiviral, antioxidant, and immunostimulating activities [6, 7]. For years, the propolis research have revealed also antitumor properties [8]. Several studies demonstrated that propolis exerted anticancer and chemopreventive properties by multiple mechanism of action [8, 9]. Some reports revealed that propolis exhibited cytotoxicity *in vitro* against many human cell lines, including colon cancer cells [10], prostate carcinoma cells [11], malignant melanoma cells [12], astroglia cells [13]. However, the molecular mechanism by which propolis exerts its cytotoxic effect on human tongue squamous cell carcinoma cell line (CAL-27) has not been studied.

*In vitro/in vivo* studies demonstrated that dietary compounds containing polyphenols are able to prevent carcinogenesis and might inhibit the growth of cancer cells [14–17]. Polyphenolic compounds abundant in green or black tea and anthocyanins occurring in black raspberries and black rice were identified as potential chemopreventive agents in human oral cancer [18–20]. Other evidences indicated that the methylated analogues of chrysin and apigenin inhibited the proliferation of human oral squamous cell carcinoma SCC-9. Methylated flavones were identified in propolis, citrus fruits and in other products applied in complementary medicine [21].

It was reported that compounds of propolis are responsible for its antitumor activity. Chrysin was found as a potent agent inducing apoptosis in many cell lines through caspase activation, suppression of anti-apoptotic proteins, such as IAPs, Akt kinase, cellular FLICE-like inhibitory protein and the inhibition of IκB kinase and NF-κB [22, 23]. Pinocembrin induced loss of mitochondrial membrane potential with releasing of cytochrome *c* and activation of caspase-3 and -9 in colon cancer cells [24]. Other results revealed that pinocembrin attenuated the cell viability of both androgen-sensitive (LNCaP) as well as androgen-independent (PC3 and DU-145) prostate cancer cell lines, with different p53 status [25]. The potency of hydroxycinnamic acids such as caffeic, ferulic, coumaric as anticancer agents, were also examined [26]. It was reported that caffeic acid induced apoptosis of lung cancer cells, through NF-κB pathway [27]. Caffeic acid also presented antiproliferative effects against colon cancer cells [28] and fibrosarcoma cancer cells [29], the latter by an oxidative mechanism.

Due to the fact that propolis is a very complex material, the effect of individual components as well as the synergistic effect of them on cancer cells should be tested. The present study focused on quantitative analysis of major flavonoids and phenolic acids in pharmaceutical formulation of propolis using GC-MS method. Previous studies reported chemical profiles and semi-quantitative analysis of ethanolic extracts of commercially available propolis samples [30]. Base on this data the most abundant phenolic compounds have been selected and submitted to quantitative analysis. In this report, for the first time the cytotoxic and pro-apoptotic activities of commercially available propolis, individual polyphenols, as well as their mixture on human tongue squamous carcinoma (CAL-27) cells were examined.

## Materials and Methods

### Materials

The silylation reagent N,O-Bis(trimethylsilyl)trifluoroacetamid (BSTFA) with 1% trimethylchlorosilane (TMSC), pyridine, dimethyl sulfoxide (DMSO), hexane, methylthiazolyldiphenyltetrazolium bromide (MTT), methyl syringate applied as internal standard (IS), pinobanksin, pinocembrin, *p*-coumaric acid, caffeic acid, ferulic acid, formaldehyde solution, albumin bovine serum (BSA), Triton^™^-100 were obtained from Sigma-Aldrich (Steinheim, Germany). Methanol for GC was purchased from POCh (Gliwice, Poland). Chrysin and galangin were purchased from Roth (Karlssruhe, Gremany). Dulbecco's Modified Eagle's Medium (DMEM), fetal bovine serum (FBS), phosphate buffered saline (PBS) and pencyllin-streptomycin (10,000 U/mL) were products of Gibco (Waltham, MA, USA). Primary antibodies anti caspase-3 (#559565), caspase-8 (#51-80851-N) and caspase-9 (#51-80861N), FITC Fluor-conjugated secondary antibody, (FITC goat anti-mouse IgG #554001, FITC goat anti-rabbit IgG #554020) were obtained from Becton Dickinson (New Jersey, USA). Hoechst 33342 (#561908) was product of ThermoFisher Scientific (USA). All chemicals and reagents used in this study were of analytical grade.

### Propolis samples

The commercial, standardized preparations of propolis were obtained from Apipol-Farma, (Myślenice, Poland) and Farmapia (Kraków, Poland). All information about an aqueous-alcoholic extracts of propolis were presented in S1 Table. The samples were stored at 4°C temperature, protected from light. The ethanolic extracts of propolis (EEP-1, EEP-2, EEP-3) were filtered through a PTFE 0.45 μm syringe filter and evaporated in rotary under reduced pressure. EEP was dissolved in DMSO (100 mg/ml) and the final concentration of DMSO in the culture medium was controlled at 0.1% (v/v) and it was found as nontoxic for cells. For GC-MS analysis, propolis sample was mixed with 20 μl of methyl syringate (100 μg/ml) used as internal standard. Then solvents were evaporated in rotary under reduced pressure. The dry residue was dissolved in 100 μl of pyridine and 100 μl of BSTFA was added into the vial. The reaction mixture was sealed and heated during 30 min at 80°C to obtain TMS derivatives. All sample procedures for GC-MS quantification were carried out in triplicate.

### Polyphenols standard solution

For cell culture experiments, the stock solutions of polyphenols were diluted with medium prior to use to obtain the desired concentration. The final concentration of DMSO on the medium was less than 0.1% that proved to have no detectable effect on cell growth. For GC-MS quantification, the stock standard solution of each polyphenolic compound was made by accurately weighing 5 mg of chrysin, galangin, pinobanksin, pinocembrin, caffeic acid, *p*-coumaric acid, ferulic acid and dissolving it in 5 ml of MeOH in a volumetric flask. The internal standard calibration curve was generated using seven data points, covering the concentration ranges in 5–500 μg/ml. All standards were injected in GC-MS instrument as a TMS derivatives.

### Cell line

Human tongue squamous cell carcinoma line CAL-27 (American Type Culture Collection, Manassas, VA, USA) was grown in DMEM (90% v/v) and fetal bovine serum (10% v/v), with the addition of penicillin (50 U/ml) and streptomycin (50 μg/ml). Culture cells were maintained at 37°C under an atmosphere of 5% CO_2_.

### Quantification of phenolic compounds by GC-MS method

Quantitative analysis of phenolic compounds was performed on gas chromatography (GC) system coupled with a quadrupole mass spectrometer (MS) instrument (Agilent Technologies, Wilmington, DE, USA). Samples were separated on a 30 m x 0.25 mm i.d., 0.25 μm film thickness, HP-5MS capillary column J&W (Agilent Technologies, Wilmington, DE, USA). The column temperature was initially held at 50°C for 10 min, and then the temperature was raised to 310°C at rate of 2°C/min, followed by an isothermal period of 10 min. Ultrapure helium with an inline oxygen and moisture trap was used as carrier gas at a flow rate of 1.2 ml/min. Aliquots of 1 μl were injected in the split (50:1) mode. The injector was kept at 280°C, MS source and MS quad temperatures were 230 and 150°C, respectively. The mass spectrometer (MS) was operated in electron impact ionization/selective ion monitoring (EI/SIM) mode. The mass fragments (m/z) used for the quantitative analysis of the polyphenols were m/z 384, **383**, and 311 for chrysin, m/z **471**, 472 and 473 for galangin, m/z 192, **296** and 369 for pinobanksin, m/z 385, 386 and **457** for pinocembrin, **219**, 396 and 397 for caffeic acid, **219**, 293 and 308 for *p*-coumaric acid, 249, 323 and **338** for ferulic acid and **254**, 269 and 284 for methyl syringate used as an internal standard (IS). The bold marked ions were used for their quantification.

### Cell viability assay

The potential cytotoxicity of studied compounds was evaluated with the MTT Carmichael colorimetric method [31]. The MTT assay is based on the reduction of the tetrazolium salt MTT to a purple formazan dye by viable cells. The amount of arising formazan is proportional to the number of living cells. CAL-27 cells (10^5^/ml) were seeded on the 96-well plates and cultured to obtain 70% confluency. Then CAL-27 cells were incubated for 24 h with varying concentrations of propolis extract: 25, 50, 100, 150 μg/ml, flavonoids (chrysin, galangin, pinocembrin): 2.5, 12.5, 25, 50 μg/ml and phenolic acids (ferulic, caffeic, *p*-coumaric): 2.5, 12.5, 25, 50, 125 μg/ml and the mixture of phenolic compounds: 2.5, 12.5, 25, 50 μg/ml for each one. After this time, the cells were washed two times with 100 μl PBS and incubated with 50 μl of MTT solution in PBS (1 mg/ml) for 1 h. Fluid was removed, and the cells were lyzed in 100 μl of DMSO with 2 μl of Sorensen's buffer (0.1 mol/l glycine with 0.1 mol/l NaCl, pH 10.5). The absorbance was measured at 570 nm wavelength using an Asys UVN 340 microplate reader (Biogenet, Józefów, Poland). The results are obtained from two independent experiments repeated six times (n = 12).

### Measurement of apoptosis by flow cytometry

AnnexinV-FITC/propidium iodide (PI) double staining assays were performed to detect apoptosis. The analysis was conducted according manufacturer’s instructions (Apoptosis Detection Kit II, BD Pharmingen, San Jose, CA, USA). CAL-27 cells were seeded in 24-well plates and grown to about 70% confluence. For dose response experiments a two concentrations of EEP (100, 150 μg/ml), flavonoids (25, 50 μg/ml), three concentration of phenolic acids (25, 50, 125 μg/ml) and polyphenolic mixture (2.5, 12.5, 25 μg/ml for each compounds) were applied to cells for 24 h. Untreated cells were used as a control. CAL-27 cells were washed by PBS and suspended in binding buffer prior to adding FITC-labeled Annexin V and PI. After 10 min incubation, suspensions were immediately analyzed by flow cytometry using a FACS Calibur machine (BD Biosciences, San Jose, CA, USA). The percentage apoptotic cells was determined.

### Measurement of apoptosis by immunofluorescence microscopy and determination of colocalization coefficient

CAL-27 cells were grown to about 70% confluence in 96-well plates and treated with EEPs (100, 150 μg/ml), flavonoids (25, 50 μg/ml), phenolic acids (25, 50, 125 μg/ml) and polyphenolic mixture (2.5, 12.5, 25 μg/ml for each compounds) for 24 h. The cells were fixed with 3.7% paraformaldehyde and permeabilized with 0.01% Triton. After blocking with 3% fetal bovine serum, cells were incubated with primary antibodies (caspase-3, caspase-8, caspase-9) at dilutions 1:500, and subsequently with FITC Fluor-conjugated secondary antibody and Hoechst. Sample were visualized with a confocal laser scanning microscope (BD Pathway 855 Bioimager). Images were subjected to the analysis of the colocalization coefficient.

### Statistical analysis

The chromatographic, cytometric, microscopic data were expressed as the mean and the standard deviation (SD) of three independent evaluations (n = 3). The qualitative analysis by GC-MS were performed using the MassHunter software. The MTT results are obtained from two independent experiments repeated six times (n = 12). The statistical significances were performed using Student’s t-test. Differences at p < 0.05 were considered to be statistically significant. All IC_50_ values were calculated from the corresponding dose inhibition curve according to their best fit shapes based on at least four or five reaction points using Stata^®^13.

## Results

### The content of phenolic acid and flavonoids in propolis extracts

The gas chromatography coupled with mass spectrometry was applied for analysis of polyphenolics in propolis extracts. The quantitative composition and phytochemical profile of this samples are presented in published work [30]. Quantitative analysis was performed for selected flavonoids and phenolic acid and data are reported in Table 1.

**Table 1:** Concentration (mg/ml) of phenolic acid and flavonoids in propolis extracts (EEP-1, EEP-2, EEP-3) by GC-MS.

Compound name	EEP-1	EEP-2	EEP-3
Chrysin	2.18 ± 0.16	1.50 ± 0.07	1.92 ± 0.04
Galangin	1.26 ± 0.02	0.50^a^	0.86^a^
Pinobanksin	0.43 ± 0.01	0.15^a^	0.24^a^
Pinocembrin	1.09 ± 0.02	0.47^a^	0.83^a^
Caffeic acid	0.93 ± 0.02	0.45^a^	0.67^a^
*p*-Coumaric acid	2.05 ± 0.13	0.59^a^	1.30 ± 0.01
Ferulic acid	0.82 ± 0.15	0.24 ± 0.02	0.82 ± 0.01
**Total**	**8.76 ± 0.51**	**3.79 ± 0.09**	**6.65 ± 0.05**

Data are expressed as mean (n = 3) ± SD,

^a^SD < 0.001

All analyzed samples contain chrysin, galangin, pinobanksin, pinocembrin, caffeic acid, *p*-coumaric acid and ferulic acid. Nevertheless, there was a meaningful variability in the concentration of the active constituents among the commercial samples of propolis. The pharmaceutical specimen indicated as EEP-1 contained the highest amount of polyphenols (8.76 mg/ml), whereas EEP-2 contained the lowest levels (3.79 mg/ml). The sample labelled as EEP-3 displayed a medium level of total polyphenols 6.65 mg/ml. Chrysin was the most significant flavonoid detected in all samples. The content of this compound ranged from 2.18 mg/ml (sample EEP-1) to 1.50 mg/ml (sample EEP-2). In the analyzed samples, the most abundant polyphenol was *p*-coumaric acid with concentration from 2.05 mg/ml to 0.59 mg/ml. S1 Fig shows the comparison of content (mg/g) of polyphenols in three different samples of commercially available EEP. The total amounts of detected polyphenols were 156.45 mg/g (sample EEP-1), 111.46 mg/g (sample EEP-2) and 114.67 mg/g (sample EEP-3).

### The cytotoxicity of EEPs, polyphenols and their mixture on CAL-27 cells

**Fig 1:**
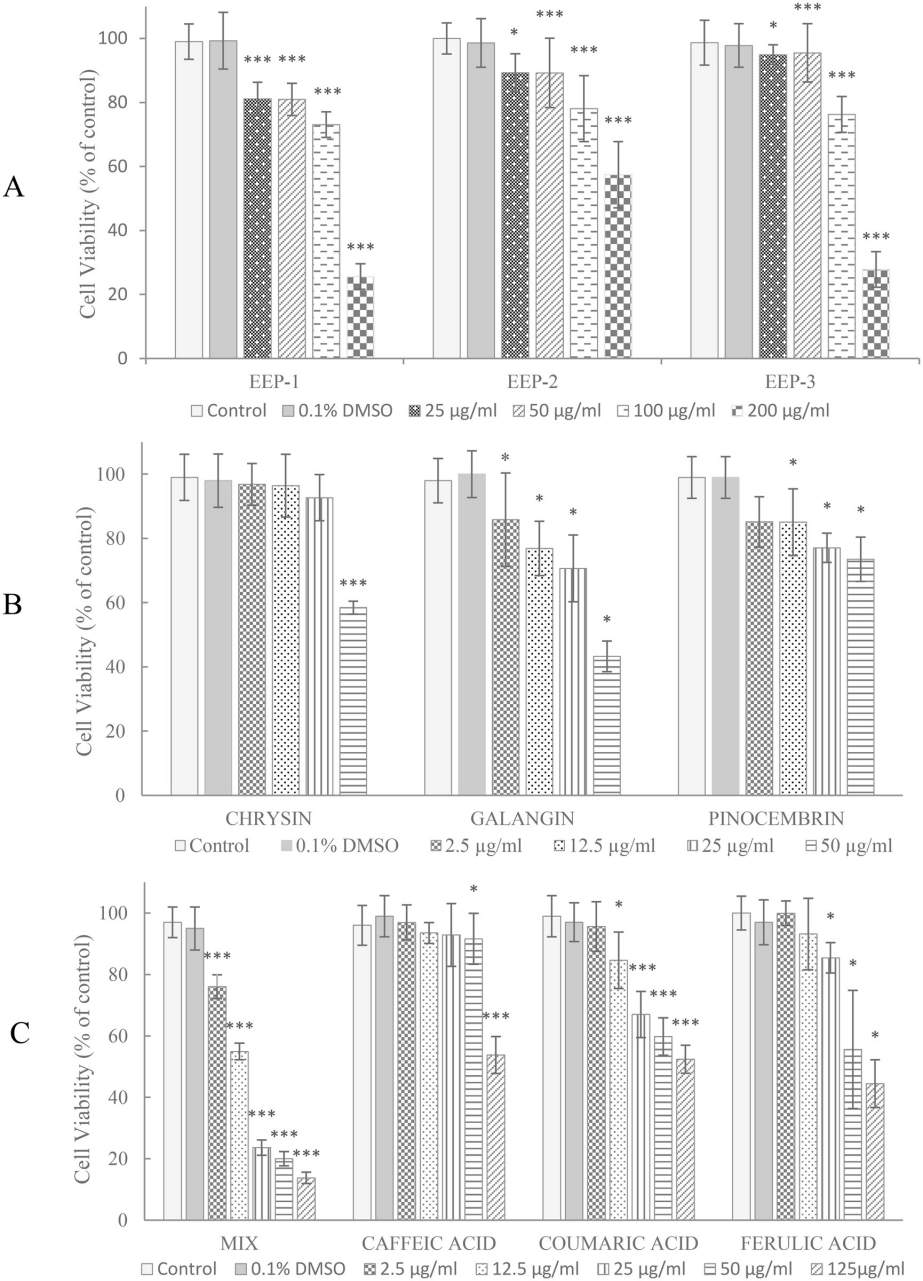
The effect of ethanolic extract of propolis (a), polyphenols, their mixture (b, c) and on viability of CAL-27 cells. The cells were treated with specified concentration of respective components for 24 h and cell viability were determined by the MTT assay. The values represent mean ± SD of two independent experiments conducted six times (n = 12). The stars indicate significance of differences (*** p < 0.001, * p < 0.05) compared to control.

The cytotoxic effect of ethanolic extract of propolis (EEP-1, EEP-2, EEP-3), polyphenols (chrysin, galangin, pinocembrin, caffeic acid, *p*-coumaric acid, ferulic acid) and mixture of this polyphenols on human tongue squamous carcinoma cell line (CAL-27) was determined by the MTT assay. The results showed that all compounds and the compounds mixture were able to induce cytotoxicity in CAL-27 cell in dose dependent manner (Fig 1).

A significant difference in growth inhibitory effects of these components on CAL-27 cells was found. The mixture of six polyphenols was more active, compared to particular constituents, with an estimated IC_50_ value of 10.7 μg/ml (Table 2). According to the IC_50_ rate of these components, the cytotoxicity for CAL-27 cells was in the order: mixture of polyphenols > galangin > chrysin > ferulic acid > caffeic acid > pinocembrin > p-coumaric acid > EEP-1 >EEP-3 > EEP-3.

**Table 2:** Concentration of polyphenolic compounds, mixture of polyphenols and ethanolic extract of propolis resulting in 50% cell viability (IC_50_) of human tongue squamous cell carcinoma CAL-27 cell line.

^a^IC_50_ ± SD (μg/ml)									
MIX	EEP-1	EEP-2	EEP-3	CHRY	GALA	PINC	FERU	CAFF	COUM
10.7 ± 2.6	159.2 ± 5.1	224.21 ± 8.9	179.0 ± 6.5	54.1 ± 6.4	44.5 ± 9.5	135.2 ± 7.4	99.6 ± 9.5	130.3 ± 7.5	139.2 ± 7.1

^a^The IC_50_ and SD were obtained via nonlinear regression and are expressed as the mean ± SD, determined from the results of the MTT assay of 2 independent experiments with 6 replicates each. The IC_50_ values are presented as the amount of component or extracts per ml of culture [IC_50_ (μg/ml) ± SD].

### Apoptotic effects of EEP, polyphenols and mixture of polyphenols on CAL-27 cells

**Fig 2:**
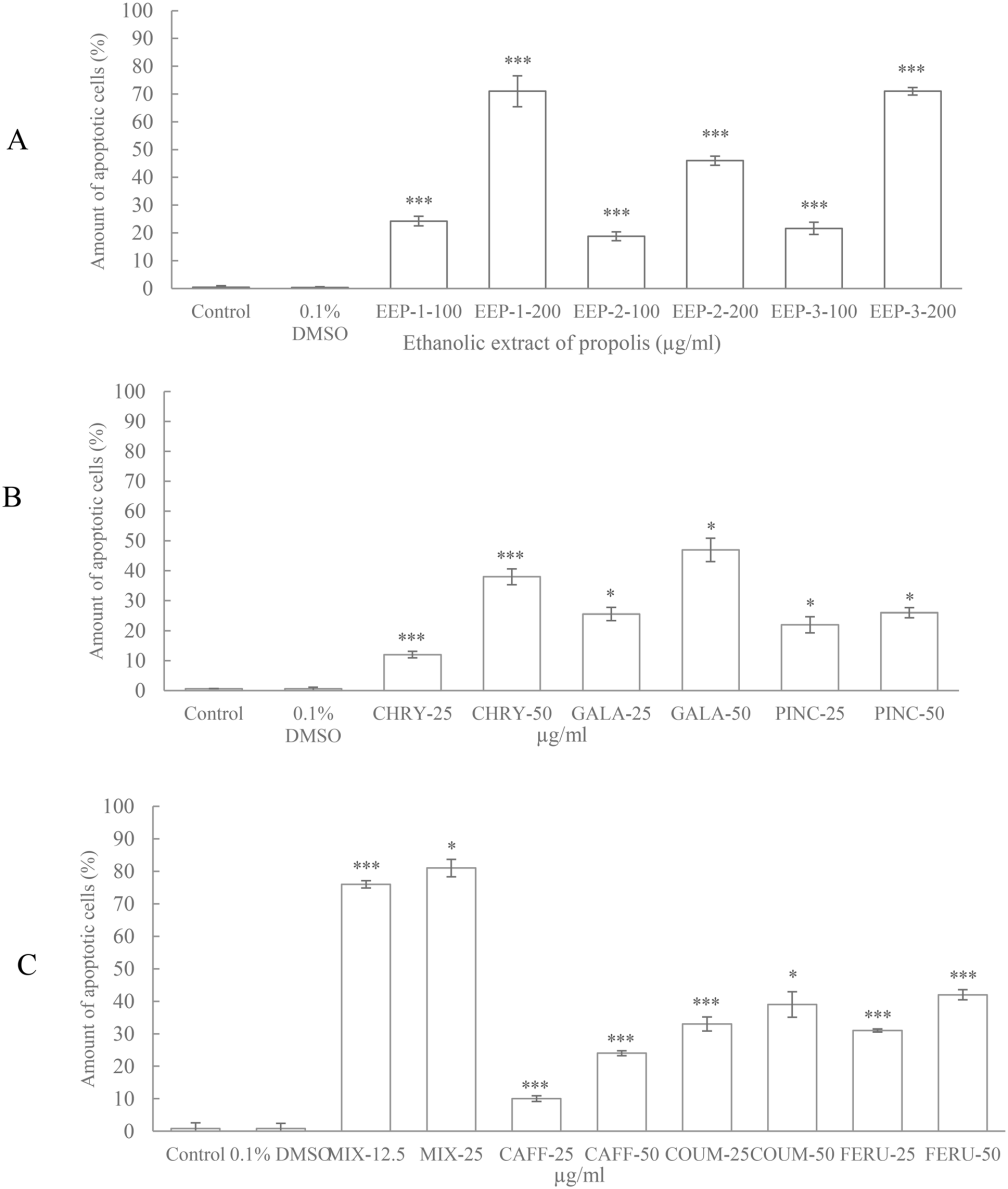
The effect of ethanolic extracts of propolis (a), polyphenols and their mixture (b, c) on the induction of apoptosis in CAL-27 cells culture, treated with respective components for 24 h. The cells were labeled with FITC-annexin V and PI. The percentages of apoptotic cells are presented. The values represent mean ± SD of three independent experiments (n = 3). An asterisk represents significant difference (*** p < 0.001, * p < 0.05) between percentage of live cells and percentage of apoptotic cells.

In view of the above-mentioned effect of the compounds on the CAL-27 cell growth, we considered apoptosis as an underlying mechanism. Flow cytometry was applied to quantify the apoptotic, alive and necrotic cells. The CAL-27 cells were exposed to two concentrations of ethanolic extract of propolis: 100 and 200 μg/ml, two concentrations of flavonoids and phenolic acid: 25 and 50 μg/ml and two concentrations of mixture of polyphenols: 12.5 and 25 μg/ml (final concentration of each compound in culture medium). Incubation of CAL-27 cells with all constituents for 24 h increased the number of apoptotic cell in various manner compared to control cells (Fig 2). The treatment of CAL-27 cell with 100 and 200 μg/ml of ethanolic extracts of propolis induced apoptosis in dose dependent manner. The weakest effect was observed in the cells treated with EPP-2.

Flavonoids at higher concentration (50 μg/ml) induced increase of apoptotic cells number in: 38% (chrysin), 47% (galangin), and 26% (pinocembrin). The induction of apoptosis by the addition of phenolic acids applied at concentration (50 μg/ml) reveled a similar number of apoptotic cell comparing to flavonoids. The caffeic acid induced apoptosis in 24% of the cells. For *p*-coumaric and ferulic acid these values were 39% and 42%, respectively. The results indicated that apoptosis was induced in most potent manner by mixture of polyphenols. The annexin V assay revealed 76 and 81% apoptotic cells in CAL-27 culture exposed to 12.5 and 25 μg/ml of the mixture. A low number of necrotic cells—below 2% (data not presented) was detected in the cells treated with flavonoids, phenolic acid, their mixtures and EEP.

### Imaging of caspases -3,-8,-9 activation

**Fig 3:**
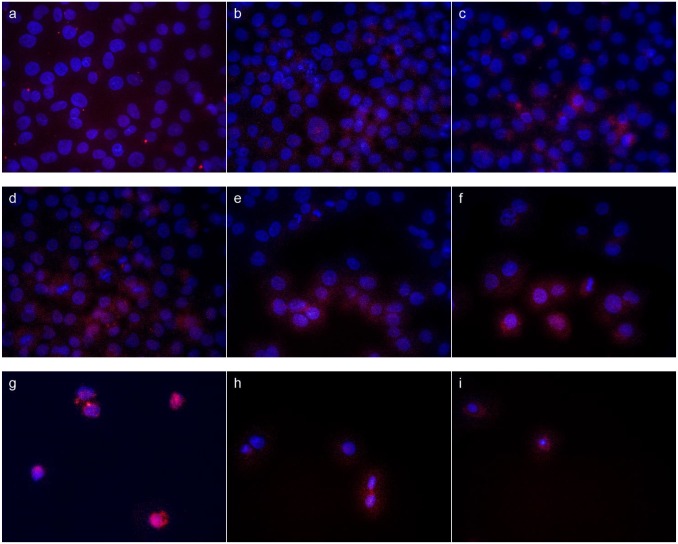
Analysis of active caspase-3, caspase-8, caspase-9 in CAL-27, treated for 24 h with EEP-1 in 200 μg/ml concentration (a, b, c), mixture of polyphenols in 12.5 μg/ml (c, d, e) and 25 μg/ml (g, h, i) concentration. Analysis were performed using anti-caspase-3 antibody (a, d, g), anti-caspase-8 antibody (b, e, h) and anti-caspase-9 antibody (c, f, i).

**Fig 4:**
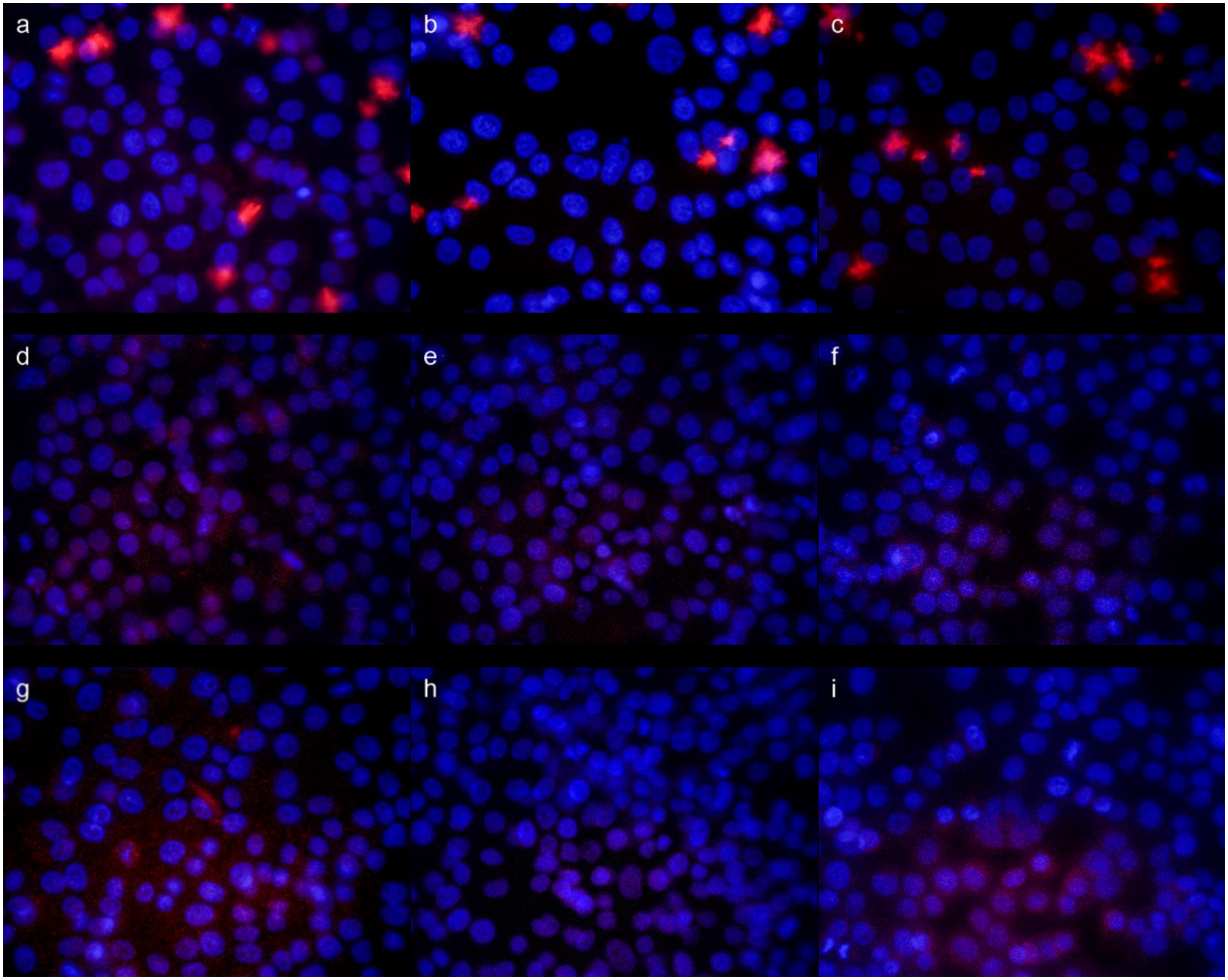
Analysis of active caspase-3, caspase-8, caspase-9 in CAL-27, treated for 24 h to chrysin (a, b, c), pinocembrin (d, e, f) and ferulic acid (g, h, i) in 50 μg/ml concentration. Analysis were performed using the anti-caspase-3 antibody (a, d, g), anti-caspase-8 antibody (b, e, h) and anti-caspase-9 antibody (c, f, i).

Three ethanolic extracts of propolis, individual components and their mixture were evaluated for ability to induce caspases -3, -8, -9. Fluorescent microscopy was applied to evaluate active forms of caspases -3, -8, -9 after 24 h treatment with the above-mentioned agents. Figs 3 and 4 present images of cell nucleus stained with Hoechst (emitted blue fluorescence) and activated caspase in cell cytoplasm, which emitted red fluorescence. All samples of EEP and mixtures of polyphenols induced activation of caspase-3, caspase-8 and caspase-9 in CAL-27 cells (Fig 3). As shown on Fig 4 activation of caspase-3, -8 and -9 was higher in CAL-27 cells incubated with chrysin (Fig 4a–4c) compared to other compounds. Among polyphenolic components, chrysin was able to induced activation of caspase-8. Galangin and *p*-coumaric had a slight effect on activation of caspase-3 and -9 in CAL-27 cells.

## Discussion

Oral cancer represent a subset of head and neck squamous cell carcinoma—a disease occurring in approximately 4% of all malignancies reported in Poland [32]. Current therapies include surgery and radiation therapy. Propolis and their constituents have been known to possess the cytotoxic effect on several cancer cell lines [10–12], but studies on human tongue cancer CAL-27 cells have not been reported. The purpose of current study was to investigate and characterize the cellular response of CAL-27 cells to propolis, polyphenols and mixture of polyphenols.

Firstly, the GC-MS method was used to quantify selected flavonoids and phenolic acid in commercially available ethanolic extracts of propolis. Quantitative analysis was performed for major components such as chrysin, galangin, pinobanksin, pinocembrin, caffeic acid, *p*-coumaric acid and ferulic acid. In our previously study, it has been shown that these compounds were the most abundant constituents identified in propolis [30].

The total analyzed polyphenols comprised 15.1% (sample EEP-1), 14.6% (sample EEP-2), 11.5% (sample EEP-3), of the dry residue content. Based on the total flavonoids levels, propolis with a content lower than 11% has been considered of low quality, while that with amount of 11–14%, 14–17% or >17% has been classified as acceptable, good and high quality, respectively [33]. Pharmaceutical specimens of hydroalcoholic extract of propolis could contain lower amount of bioactive compounds comparing to lab-made extracts due to processing which consists of many steps. Raw propolis submitted to decoction and maceration proceeding, with a sample-to-solvent ratio 1:10 (w/v) and EtOH as the extraction solvent [34]. Nonetheless, the production of pharmaceutical propolis formulation is standardized and guarantees the high quality of product.

In our study, we examined the cytotoxic effects of EEP, flavonoids, phenolic acid and mixture of polyphenols on human tongue cancer cells (CAL-27). It was presented that all of agents were able to induce cytotoxicity in CAL-27 cells in a dose-dependent manner, and the effectiveness of these factors was in the order of mixture of polyphenols > galangin > chrysin > ferulic acid > caffeic acid > pinocembrin > *p*-coumaric acid > EEP-1 > EEP-3 > EEP-2. The mixture of polyphenolic compounds included chrysin, galangin, pinocembrin, caffeic, *p*-coumaric and ferulic acid, was found as the strongest agent exerted growth inhibitory effects on CAL-27 cells. It is suggested that synergistic effects of polyphenols are responsible for their cytotoxicity. In addition, compared structural characteristics of flavonoids and phenolic acids, it was suggested that their cytotoxic activities on CAL-27 cells might be dependent on the total number of hydroxyl groups in the molecule. Galangin with three -OH groups showed the most potent cytotoxicity (IC_50_ = 54.1 μg/ml) in CAL-27 cells culture. The relationship between the chemical structure of phenolic compounds and their anticancer activities, had been observed in colorectal carcinoma cells [35] and neuroblastoma cells [36]. Flavonoids and phenolic acid are considered to be antioxidants and inhibition of cell growth could depend on the capacity of these compounds to process as free radical scavengers [35].

The inhibition of cancer cell proliferation by anticancer agents could undergo at least partially through apoptosis. In present study, induction of apoptosis by selected constituents was quantified by flow cytometry after labeling the cells with FITC-annexin V/PI. Our study demonstrated that the treatments with all agents resulted in induction of apoptosis of CAL-27 cells. The findings showed that the potency of EEP, mixture and their components on induction of apoptosis were similar to their potency on growth inhibition. These outcomes clearly suggest that all agents inhibit the growth of tongue cancer via apoptosis.

Apoptosis is characterized by various biochemical criteria, including changes in mitochondrial membrane permeability, caspase activation, internucleosomal DNA cleavage and release of intermembrane space mitochondrial proteins. Apoptotic cell death also includes a series of morphological modifications such as formation of apoptotic bodies, chromatin fragmentation, nuclear and cytoplasmic condensation [37]. In the present study, the induction of apoptosis by EEP, polyphenols and their mixture in human tongue cancer cells was confirmed by triggering activities of caspase-3, -8, -9. There are two signaling pathways of caspase-mediated apoptosis, the mitochondrial (intrinsic pathway) and the death receptor (extrinsic pathway). The mitochondrial pathway mediated by cleaved caspase-9 is activated in response to extracellular cues and internal insults such as DNA damage [38]. In this study, an expression of caspase-9 was measured in CAL-27 cells treated with all agents. Furthermore, the increase of cleaved capase-9 conducted to subsequent activation of caspase-3 (Figs 3 and 4). Confocal laser microscope analyses indicated that EEPs, mixtures of polyphenols and chrysin increased the active form of caspase-3. These results demonstrated that these agents induced apoptosis by mitochondrial and death receptor pathways. We also determined that pinocembrin and ferulic acid induced apoptosis of CAL-27 cells by the mitochondrial pathway.

Many studies have demonstrated that the mechanisms of action of polyphenols involves scavenging of free radicals, regulation of gene expression, induction of cell cycle arrest, apoptosis and stimulation of the immune system [39]. Our report suggests that the synergistic effects of polyphenols in propolis are responsible for their potential anticancer activities. Therefore, ethanolic extracts of propolis could be considered as a chemopreventive agent in a human tongue squamous cell carcinoma.

## Supporting Information

S1 Fig Comparison of content (mg/g) of phenolic compounds in three different samples of commercial available of propolis. (TIF)

S1 Table Characteristic of commercial preparations containing the hydroalcoholic extracts of propolis according to information claimed on the label. (DOCX)
