# Supplementary figures and images for: Proapoptotic Activity of Propolis and Their Components on Human Tongue Squamous Cell Carcinoma Cell Line (CAL-27)
Source: PLoS One. 2016 Jun 9;11(6):e0157091. doi: 10.1371/journal.pone.0157091 (PMC4900600; doi:10.1371/journal.pone.0157091)

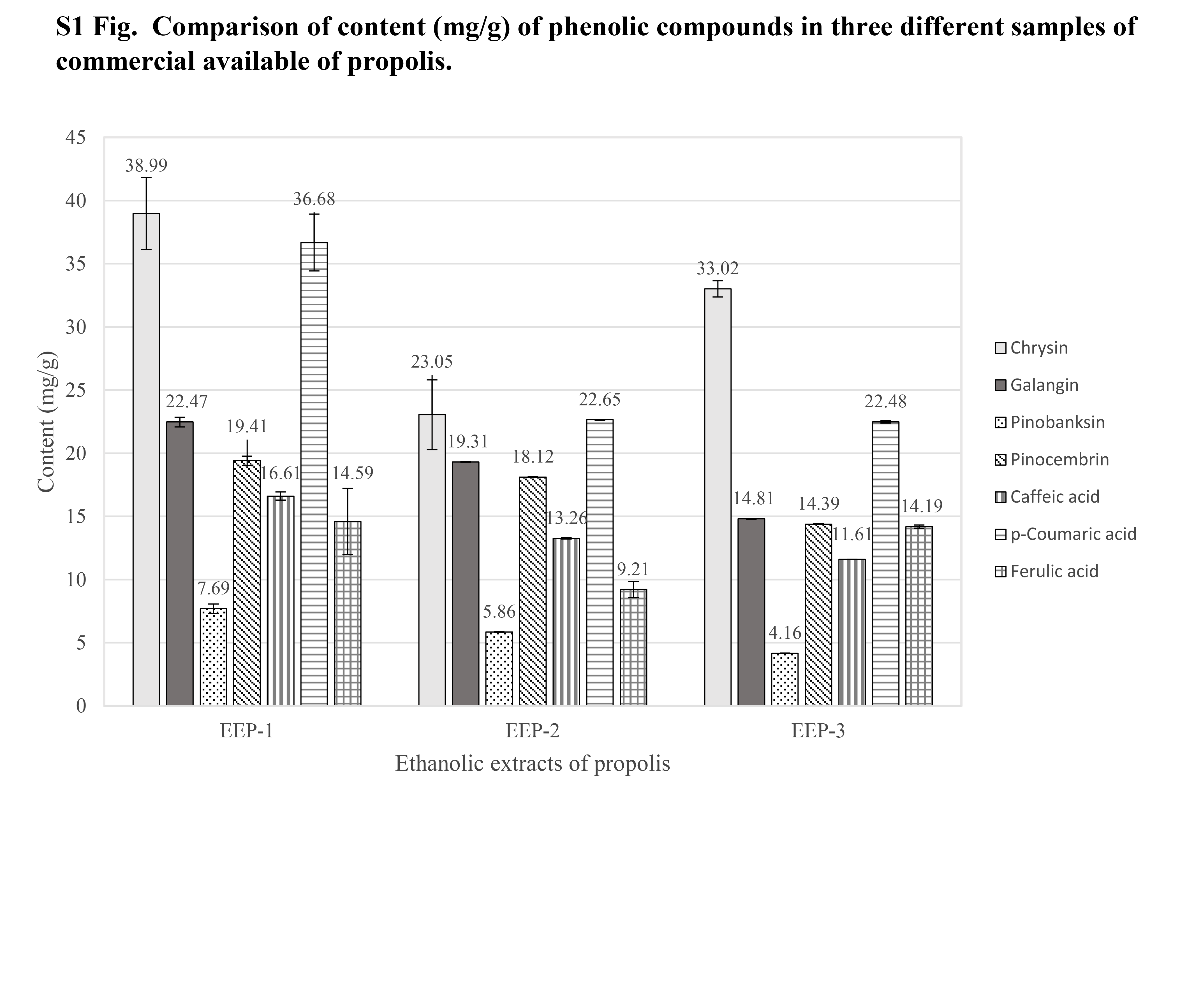

Supplement: S1 Fig — (TIF) [file pone.0157091.s001.tif]
