# Supplementary material for: Proapoptotic Activity of Propolis and Their Components on Human Tongue Squamous Cell Carcinoma Cell Line (CAL-27)
Source: PLoS One. 2016 Jun 9;11(6):e0157091. doi: 10.1371/journal.pone.0157091 (PMC4900600; doi:10.1371/journal.pone.0157091)
Supplement: S1 Table — (DOCX) [file pone.0157091.s002.docx]

**S1 Table.** **Characteristic of commercial preparations containing the hydroalcoholic extracts of propolis according to information claimed on the label.**

| **Sample** | **Manufacturer** | **Lot no.** | **Other information** | |
| --- | --- | --- | --- | --- |
|  |  |  | **Concentration** | **EtOH content** |
| **EEP-1** | Farmapia | 11041 | 58 mg/mL | 60% |
| **EEP-2** | Apipol Farma | 70611 | 25 mg/mL | 93% |
| **EEP-3** | Farmapia | 12068 | 58 mg/mL | 60% |
